# Gene regulation induced in the C57BL/6J mouse retina by hyperoxia: a temporal microarray study

**Published:** 2008-10-31

**Authors:** Riccardo Natoli, Jan Provis, Krisztina Valter, Jonathan Stone

**Affiliations:** 1 ARC Centre of Excellence in Vision Science, The Australian National University, Canberra, Australia; 2 Visual Sciences Group, School of Biology, The Australian National University, Canberra, Australia; 3 Save Sight Institute and Discipline of Physiology, University of Sydney, Sydney, Australia

## Abstract

**Purpose:**

Hyperoxia is specifically toxic to photoreceptors, and this toxicity may be important in the progress of retinal dystrophies. This study examines gene expression induced in the C57BL/6J mouse retina by hyperoxia over the 14-day period during which photoreceptors first resist, then succumb to, hyperoxia.

**Methods:**

Young adult C57BL/6J mice were exposed to hyperoxia (75% oxygen) for up to 14 days. On day 0 (control), day 3, day 7, and day 14, retinal RNA was extracted and processed on Affymetrix GeneChip^®^ Mouse Genome 430 2.0 arrays. Microarray data were analyzed using GCOS Version 1.4 and GeneSpring Version 7.3.1. For 15 genes, microarray data were confirmed using relative quantitative real-time reverse transcription polymerase chain reaction techniques.

**Results:**

The overall numbers of hyperoxia-regulated genes increased monotonically with exposure. Within that increase, however, a distinctive temporal pattern was apparent. At 3 days exposure, there was prominent upregulation of genes associated with neuroprotection. By day 14, these early-responsive genes were downregulated, and genes related to cell death were strongly expressed. At day 7, the regulation of these genes was mixed, indicating a possible “transition period” from stability at day 3 to degeneration at day 14. When functional groupings of genes were analyzed separately, there was significant regulation in genes responsive to stress, genes known to cause human photoreceptor dystrophies and genes associated with apoptosis.

**Conclusions:**

Microarray analysis of the response of the retina to prolonged hyperoxia demonstrated a temporal pattern involving early neuroprotection and later cell death, and provided insight into the mechanisms involved in the two phases of response. As hyperoxia is a consistent feature of the late stages of photoreceptor degenerations, understanding the mechanisms of oxygen toxicity may be important therapeutically.

## Introduction

Retinal dystrophies are a diverse group of conditions with the common feature of photoreceptor degeneration. Many have a genetic basis [1] but environmental factors, such as oxygen [2] and ambient light [3–6], play a role in determining their phenotype. This study concerns the impact of excess oxygen on nondegenerative photoreceptors in the C57BL/6 mouse. Oxygen levels in the outer (photoreceptor) layers of retina vary more widely than in the inner layers, or in other regions of the central nervous system [7], because of the lack of autoregulation in the choroidal circulation [8]. Hyperoxia is specifically and directly toxic to photoreceptors [9–13], and oxygen levels in outer retina rise in probably all forms of retinal degeneration, as the photoreceptor population is depleted [14–16]. As a consequence, hyperoxia may play a role in the progression of photoreceptor degenerations, whatever the initial cause.

The genes that regulate the response of photoreceptors to hyperoxia are not well understood. The effect of hyperoxia on several housekeeping genes [17] and the early response of the protective *Oxr1* gene [18] have been described, and a strong genetic determinant of resistance to hyperoxic toxicity has been traced to chromosome 6 in the A/J mouse strain [19]. However, to date no study has described global changes in retinal gene expression of genes in response to sustained hyperoxia. This paper describes changes in gene expression over a 14-day period previously demonstrated as necessary for the induction of photoreceptor death in the C57BL/6J mouse [10,19].

## Methods

### Rearing conditions

C57BL/6J mice were used because of the vulnerability of their photoreceptors to hyperoxia. Mice were raised in dim (5 lux) cyclic illumination (12 h:12 h light-dark cycle). These levels of lighting were maintained during exposure to hyperoxia. Three-month-old adult mice were placed in a clear Plexiglass chamber and exposed to normoxia (control – 0 days) or 75% oxygen for 3, 7, or 14 days. Oxygen levels were maintained using a Oxycycler feedback-controlled device (Biospherix, Lacona, NY). Animals were treated in accordance with the ARVO Statement for the Use of Animals in Ophthalmic and Vision Research.

### RNA extraction and analysis

RNA extraction was performed using a combination of TRIzol Reagent (Cat# 15596–026; Invitrogen, Carlsbad, CA) and RNAqueous-micro kit (Cat# 1931; Ambion, Foster City, CA). TRizol was used to extract the RNA, and the RNAqueous kit was used to purify and DNase-treat the RNA.

Animals were sacrificed by cervical dislocation and the retinas removed. For each time point (0, 3, 7, and 14 days), 12 retinas (from three males and three females) were pooled and placed into a 1.5 ml tube containing 200 µl of TRIzol and homogenized on ice. Following homogenization, a further 660 µl of TRIzol and 160 µl of chloroform were added to the tube. The tube was vortexed for 20 s and allowed to stand for 7 min at room temperature. The tubes were centrifuged at 13,000x g for 10 min at 4 °C. The supernatant was then removed and placed into a clean 1.5 ml tube with half its volume of 100% ethanol. The tube was vortexed briefly before its contents underwent purification (Part C. RNA Isolation, Ambion Protocol) and DNase treatment (Part D, Ambion Protocol) as detailed in the RNAqueous-micro kit manual.

Purified DNAase-treated RNA was analyzed on a ND-1000 spectrophotometer (Nanodrop Technologies, Wilmington, DE) and a 2100-Bioanalyzer (Agilent Technologies, Santa Clara, CA), to determine the quantity and purity of the sample. RNA samples were used only if the 260/280 ratio was above 1.9 and the RIN (RNA integrity number) was greater than 8.5.

### Microarray analysis

To study the changes in gene expression induced by hyperoxia, we used 8 Affymetrix (Santa Clara, CA) Mouse Genome 430 2.0 arrays. These microarrays contain over 39,000 transcripts representing 34,000 genes. Labeling, hybridization, washing, and scanning of the microarray were performed at the ACRF Biomolecular Resource Facility at the John Curtin School of Medical Research, Australian National University, following the manufacturers’ specifications (Affymetrix). The arrays were scanned on the GCS 3000 Affymetrix high resolution scanner and analyzed using the GeneChip Operating Software v1.4 (GCOS; Affymetrix) and GeneSpring v 7.3.1 (Agilent Technologies). Normalization was performed using the Microarray Suite 5 (MAS5) algorithm and only gene expression levels with statistical significance (p<0.05) were recorded as being “present” above background levels. Genes with expression levels below this statistical threshold were considered as “absent.” To determine the changes in gene expression between control and hyperoxia treated retinas, we treated the control RNA as a baseline control and expressed changes relative to that control. Increases were determined for genes that were considered present or absent in the control and present in the hyperoxia sample; decreases were determined for genes that were present in the control sample and present or absent in the hyperoxia sample. Only fold changes of two or greater were considered significant.

Replicate data were consolidated into groups based on time exposed to hyperoxia (0, 3, 7 and 14 days) and organized using the hierarchical clustering, ANOVA (ANOVA) and gene ontology (GO) functions in the GeneSpring software.

Hierarchical clustering was done using the clustering function (condition tree) in GeneSpring. All gene expression levels were used as a gene list and experiments were organized by both individual samples (to test sample reproducibility) as well as groups (time exposed to hyperoxia). ANOVA analysis was performed to search for genes which varied most prominently across the different groups. Microarray were analyzed using a parametric test and the cross gene error model with a p-value cut-off set to 0.05. GO searches for biologic processes were also performed, using data obtained from initial GCOS twofold analysis to identify genes involved in the response to stress and apoptosis.

### qPCR

RNA for qPCR was handled in the same way as RNA extracted for the GeneChip^®^ experiments, with the exception of group numbers. Three biologic groups were used, each containing three animals at each time point. Superscript III and the accompanying standard protocol (Invitrogen) were used to convert 1 µg of retinal RNA to cDNA. TaqMan^®^ probes (Applied Biosystems, Foster City, CA) were used to assess the validity of gene expression changes identified in the microarray experiment (Table 1).

**Table 1:** List of TaqMan probes.

**Gene symbol**	**Gene name**	**Catalogue**
*LIN7B*	Lin-7 homolog B	Mm00457059_m1
*EDN2*	Endothelin 2	Mm00432983_m1
*SIVA1*	Apoptosis regulatory protein Siva	Mm00834449_g1
*BCL3*	B-cell leukemia/lymphoma 3	Mm00504306_m1
*OPN1SW*	Short-wave-sensitive opsin	Mm00432058_m1
*OPN1LW + OPN1MW*	Medium/long-wave-sensitive opsin	Mm00433560_m1
*RHO*	Rhodopsin	Mm00520345_m1
*C3*	Complement component 3	Mm00437858_m1
*DMD*	Dystrophin, muscular dystrophy	Mm00464475_m1
*PRPF3*	Pre-mRNA processing factor 3 homolog	Mm00510550_m1
*GADD45β*	Growth arrest and DNA-damage-inducible 45 beta	Mm00435123_m1
*HIF1α*	Hypoxia inducible factor 1, alpha subunit	Mm00468869_m1
*GAPDH* (Control)	Glyceraldehyde-3-phosphate dehydrogenase	Mm99999915_g1

List of all TaqMan probes used in this study to validate the microarray data. GAPDH was used as the normalizing control gene for all comparisons.

TaqMan^®^ probes were applied following manufacturer’s instructions with the Gene Expression Master-Mix (Applied Biosystems), and the qPCR was performed on a Rotor-Gene 3000 and analyzed using the Rotor-Gene 6 software (Corbett Robotics, Mortlake, NSW, Australia). For each biologic sample, measurements were performed in duplicate and the C_t_ (cycle threshold) means were used to determine fold-change, using the Pfaffl Equation [20]. *GAPDH* primers were employed to amplify a reference standard product that shows no regulation by hyperoxia in this study. The fold change was expressed relative to control (day 0 hyperoxia) values and normalized to the reference gene (*GAPDH*).

## Results

### GeneChip^®^ data: preliminary analyses

#### Quality Assessment

Samples were analyzed for validity against a set of criteria detailed in the Affymetrix Data Analysis Manual. All samples met the specified criteria (Table 2). Specifically, *GAPDH* 5′/3′ ratios were less than 3, background values were between 20 and 100, the noise (RawQ), scale factors and percent present values were similar in the replicates, and the percent present value was estimated to be 50% for all samples.

**Table 2:** Measures of sample and data quality.

**Experiment #1**	**Day 0**	**Day 3**	**Day 7**	**Day 14**
GAPDH 5′/3′ ratios	1.03	0.87	0.81	2.16
Background	43.41	55.04	37.47	39.53
Noise (rawQ)	1.34	1.69	1.16	1.19
Scale factor	1.765	1.524	2.785	2.586
Percent present	53.4	55	47.8	48
**Experiment #2**	**Day 0**	**Day 3**	**Day 7**	**Day 14**
GAPDH 5′/3′ ratios	1.11	0.79	1.03	2.44
Background	43.77	53.3	42.76	40.21
Noise (rawQ)	1.36	1.67	1.31	1.24
Scale factor	1.68	1.533	1.808	2.376
Perecent present	54.7	54.9	51.4	47.5

The quality of each chip was evaluated using standardized criteria including 5′/3′ ratio of GAPDH, background, noise (rawQ), scale factor, and percent present. All values met the required criteria.

#### Hierarchical clustering analysis

**Figure 1:**
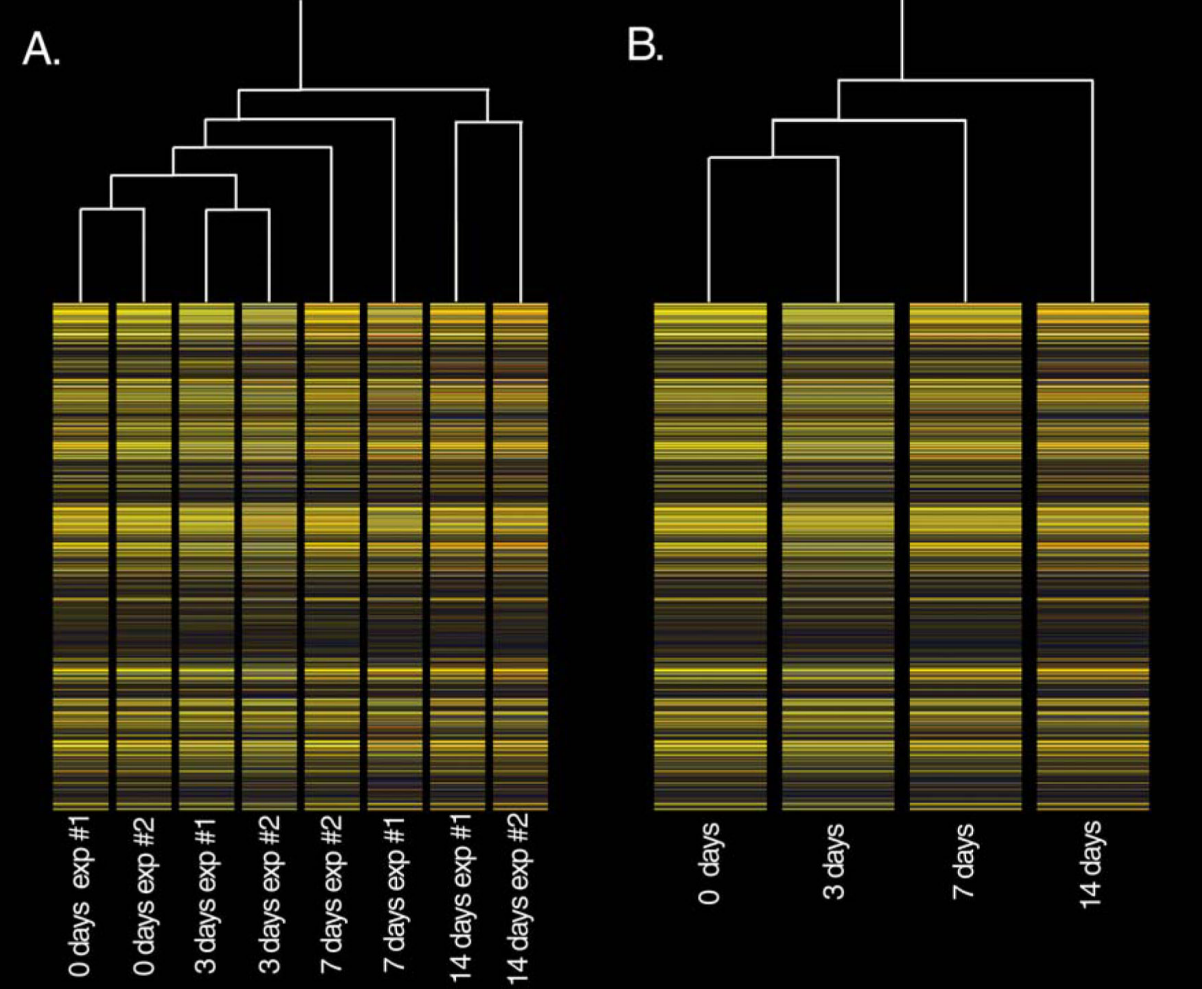
Hierarchical clustering diagrams showing individual replicates (**A**) and pooled data (**B**). Panel **A** shows strong replicate clustering at all time points except 7 days, while Panel **B** shows that the change of global gene expression is continual with the exposure to oxygen.

The hierarchical clustering analysis (Figure 1A) showed strong replicate clustering at day 0, day 3, and day 14. The day 7 replicates did not cluster closely, however, suggesting biologic variability at this time point. When the replicates were combined (Figure 1B), the clustering analysis showed that, overall, gene expression diverged steadily from control values with time of exposure to hyperoxia, with the day 14 sample furthest separate from the control.

#### Analyses of changes in gene expression

Four approaches to the analysis of gene expression changes induced by hyperoxia are summarized in Table 3. The first approach tallied the genes for which expression had increased or decreased by twofold or greater. The numbers of genes whose expression change met this criterion increased steadily during the hyperoxic treatment—from 1,177 at day 3 to 2,393 at day 7 to 3,102 at day 14. Decreases in expression were prominent at all three time points and became more prominent with time, with the decrease:increase ratio rising from 1.3 at day 3, when photoreceptors were still resistant to hyperoxia, to 2.3 at day 14, when photoreceptor degeneration was under way.

**Table 3:** Numbers of genes changing expression with hyperoxia.

	**Time in hyperoxia**		
	**3 days**	**7 days**	**14 days**
Increased expression	509	967	933
Decreased expression	668	1426	2169
Total genes changing expression	1177	2393	3102
ANOVA	18	19	95
Retinal Disease Genes	0	0	15
GO: Response to stress	58	108	196
GO: Apoptosis-related	45	81	101

The number of genes regulated by hyperoxia, over a 14 day exposure, with a change of ±2 fold or greater is shown. The data were further analyzed and divided into catagories based on several criteria, including gene increases (first row), decreases (second row), total number of gene changes (third row), number of genes with a p<0.05 (ANOVA; fourth row) and genes associated with human retinal diseases (fifth row). Using Gene Ontology (GO), those genes associated with stress (sixth row) and apoptotic cell death (seventh row) were also determined.

The second approach was an ANOVA of the genes identified by the twofold change criterion. This provided a list of genes for which the difference from control values was significant (p<0.05), for each time point. The number of genes significantly altered increased with exposure hyperoxia, particularly at day 14 (Table 3).

In the third approach, we compared the list of genes which met the twofold criterion with the list of genes in which mutations cause photoreceptor dystrophy in humans (RetNet database). This comparison identified 15 retinal disease genes that were significantly regulated by hyperoxia at day 14.

In the fourth approach, we used the GO function of the GeneSpring software to characterize functional groups of the genes in the twofold criterion list. Prominent among those functional groupings were genes associated with the response of tissue to stress, and with apoptotic cell death.

### qPCR validation

Six genes from the list isolated by ANOVA analysis of the GeneChip^®^ results (Appendix 1) were selected for analysis by qPCR: B-cell leukemia/lymphoma 3 (*BCL-3*; apoptosis-related), Complement component 3 (*C3*; complement cascade), Glial fibrillary acidic protein (*GFAP*; response to stress), Endothelin 2 (*Edn2*; vasoconstriction), Lin-7 homolog B (*Lin7b*; channel maintenance), and Growth arrest and DNA-damage-inducible 45 beta (*Gadd45b*; apoptosis). They represent several functional groups and were strongly regulated by hyperoxia. In addition, three genes were chosen from the twofold criterion list as genes of interest in hyperoxic stress:hypoxia-induced transcription factor (*HIF1*α), free radical scavenger (*Oxr-1* [18]) and apoptosis-inducing (*Siva*). Three other genes were chosen for study, because they showed changes in expression of <2 fold (no change) in the GeneChip data: rhodopsin, L/M opsin, and S opsin. These genes were chosen to validate a group of known retinal expressed genes (opsins) that exhibited no change in this study. Three genes were chosen from a group of human retinal disease genes (see Table 4): *C3* (already included), dystrophin, muscular dystrophy (*DMD*) and PRP3 pre-mRNA processing factor 3 (*Prpf3*). GAPDH was included, as a gene with constant expression during hyperoxia.

**Table 4:** Genes involved in human retinal disease that were found to be regulated by 14-day hyperoxia.

**Probe ID**	**Gene symbol**	**Gene title**	**Fold change**
1439083_at	*AHI1*	Abelson helper integration site	−2.805
1447156_at	*CHM*	Choroidermia (Chm), mRNA	−4.097
1423954_at	*C3*	Complement component 3	10.41
1421451_at	*CRB1*	Crumbs homolog 1 (Drosophila)	−2.381
1417307_at	*DMD*	Dystrophin, muscular dystrophy	−5.376
1448665_at	*DMD*	Dystrophin, muscular dystrophy	−2.298
1446156_at	*DMD*	Dystrophin, muscular dystrophy (DMD), mRNA	−4.319	
1438251_x_at	*HTRA1*	HtrA serine peptidase 1	2.04	
1445740_at	*MASS1*	Monogenic, audiogenic seizure susceptibility 1, mRNA (cDNA clone IMAGE:5050650)	−2.816	
1451062_a_at	*PEX2*	Peroxin 2	−2.493	
1446144_at	*PEX2*	Peroxin 2	−2.47	
1437443_at	*PXMP3*	Peroxisomal membrane protein 3, mRNA (cDNA clone MGC:11449 IMAGE:3964491)	−2.192	
1421381_a_at	*COL9A1*	Procollagen, type IX, alpha 1	−2.164	
1418599_at	*COL11A1*	Procollagen, type XI, alpha 1	−2.151	
1442326_at	*PCDH15*	Protocadherin 15	−3.879	
1457390_at	*PRPF3*	PRP3 pre-mRNA processing factor 3 homolog (yeast) (Prpf3), mRNA	−5.155	
1439635_at	*RGS9*	Regulator of G-protein signaling 9	−2.126	
1427467_a_at	*RPGR*	Retinitis pigmentosa GTPase regulator	−2.808	
1451785_at	*RPGRIP1*	Retinitis pigmentosa GTPase regulator interacting protein 1	−2.651	
1431357_a_at	*RPGRIP1*	Retinitis pigmentosa GTPase regulator interacting protein 1	−2.547	
1454231_a_at	*Rpgrip1*	Retinitis pigmentosa GTPase regulator interacting protein 1	−2.39	
1456449_at	*Rpgrip1*	Retinitis pigmentosa GTPase regulator interacting protein 1, mRNA (cDNA clone IMAGE:4504262)	−21.15	
1448411_at	*Wfs1*	Wolfram syndrome 1 homolog (human)	2.07	

Listed are human retinal disease genes whose expression was found to change by greater than twofold after a 14-day exposure to hyperoxia. List was generated from RetNet database. Red underline marks genes validated by qPCR.

**Figure 2:**
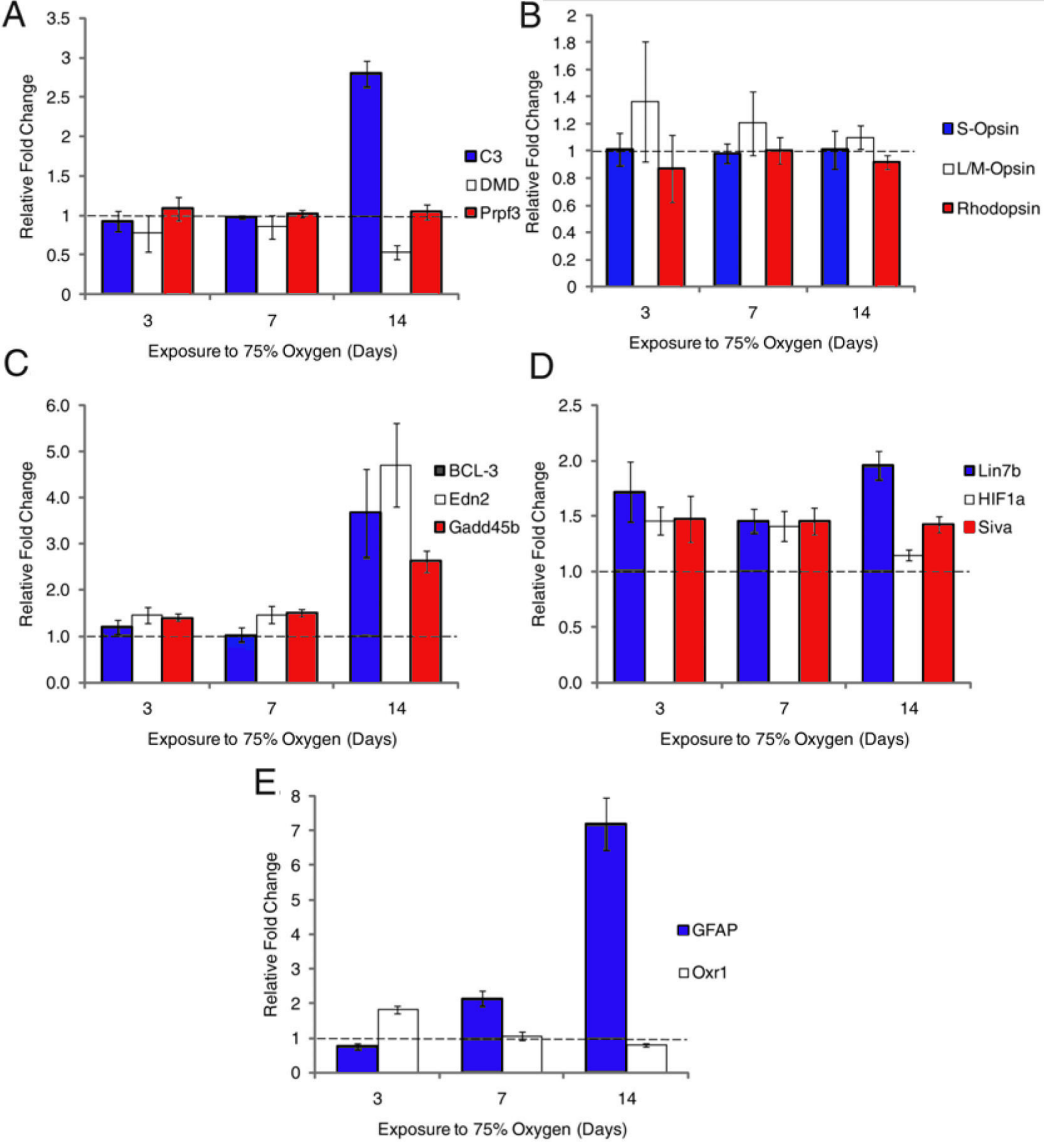
Assessment by qPCR of expression changes in genes identified by microarray analysis as hyperoxia-regulated. As noted for Table 5, these data validate trends in the Genechip data. **A:** Validation of human retinal disease genes showing upregulation of C3, downregulation of DMD and no change to Prpf3. **B:** Control genes (opsins) which showed no change in expression. **C-E:** Additional sets of genes assessed by qPCR. **C** shows a group of three genes upregulated at 14 day. **D** shows a group upregulated at all time points; **E** shows contrasts a gene (Oxr1) upregulated at 3 day, with GFAP, which is maximally upregulated at 14 day. For each gene, the fold change in expression was determined using 0 day expression as control and normalizing the data to the housekeeping gene GAPDH. Fold changes below the dashed line indicate expression decrease while above the line indicate expression increase. Error bars show standard error of the mean.

For each of these 15 genes, the fold changes observed by qPCR (Figure 2) and in the GeneChip analysis are shown in Table 5. The magnitude of the change varied considerably between the two techniques but, with three exceptions, the direction of the change was the same in the two assessments. The three exceptions were *C3* at day 7 (correlated at day 3 and day 14 but diverged at day 7), *HIF1*α (diverged at day 14), and *Prpf3* (no correlation with GeneChip data).

**Table 5:** Comparison of Genechip and qPCR data.

** ** **Gene**	**qPCR**			**GeneChip**		
	**Day 3**	**Day 7**	**Day 14**	**Day 3**	**Day 7**	**Day 14**
*BCL-3*	1.21	1.03	3.66	NC	NC	4.1
*C3*	−1.11	1	2.8	NC	−6.21	10.42
*Edn2*	1.46	1.48	4.71	2.93	NC	13.15
*Gadd45b*	1.37	1.77	3.35	NC	NC	4.54
*GFAP*	−1.33	2.14	7.2	NC	2.31	16.64
*HIF1a*	1.5	1.4	1.1	3.48	NC	−3.37
*Lin7b*	1.72	1.45	1.96	NC	NC	4.39
*L/M-opsin*	1.4	1.2	1.1	NC	NC	NC
*Oxr1*	1.83	1.08	−1.25	3.3	NC	−2.6
*Rhodopsin*	−1.11	1	−1.11	NC	NC	NC
*Siva*	1.47	1.45	1.42	NC	NC	2.3
*S-opsin*	1	1	1	NC	NC	NC
*DMD*	−1.25	−1.23	−2	NC	NC	−5.38, −2.3, −4.13
*Prpf3*	1.1	1	1	NC	NC	−5.2
GAPDH*	1	1	1	NC	NC	NC

Generally, genes showed the same trend in regulation in the qPCR and microarray experiments, but not necessarily by the same fold change. No change (NC) refers to a gene whose expression was below twofold for the microarray experiment and therefore the results were not statistically significant. The asterisk indicates a housekeeping gene. Validation, using qPCR, of genes linked to human retinal diseases were found to have differential expression at day 14 exposure to hyperoxia. Multiple entries indicate multiple probe identification (ID)’s from the GeneChip® analysis. Note that *Prpf3* did appear to change when tested using qPCR.

### Functional groupings of oxygen-induced genes

From the genes identified in this study to be regulated by hyperoxia, many functional groupings are possible. Three are considered in more detail here because of their relevance to photoreceptor degeneration

### Human retinal disease genes

The 15 retinal disease genes that were upregulated at day 14 exposure are shown in Table 4. Only one of the 15 is known to be retina specific: Regulator of G-protein signaling 9 (*RGS9*) is a photoreceptor specific member of a family of proteins that deactivate transducins. The other genes are more ubiquitous, although some, such as *DMD* and Retinitis pigmentosa GTPase Regulator (*RPGR*), may have retina-specific isoforms..

### Stress-related genes

**Figure 3:**
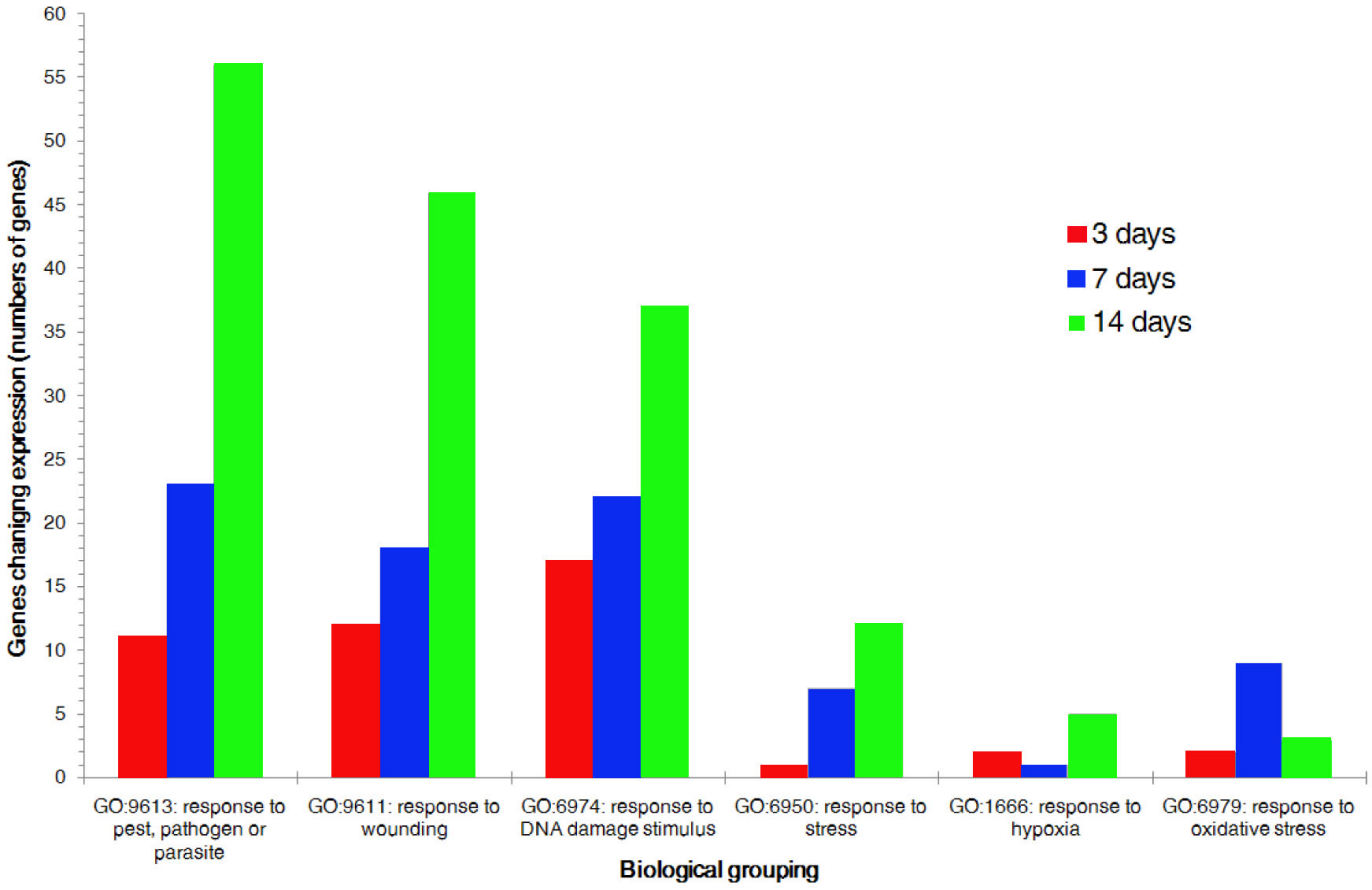
Genes for which expression was up- or downregulated by twofold or more. These were separated into the subgroupings of the gene ontology (GO) category “response to stress.” The number of genes regulated by hyperoxia increased with exposure and was maximal at day 14 in all subgroupings, except oxidative stress, which was maximal at day 7.

As noted, the number of stress-related genes whose expression was significantly changed by hyperoxia increased from 58 at day 3 exposure to 196 at day 14 (Appendix 1). Figure 3 breaks the GO-identified response-to-stress genes into biologic subgroupings.

In all subgroupings except oxidative stress, the number of genes regulated by hyperoxia increased with exposure, and was maximal at day 14. Within this overall trend, however, there was a significant shift in the subgroups regulated. At day 3, for example, the most numerous subgroup was of genes relating to DNA damage; at day 14, by contrast, genes relating to damage (“wounding” and “pest, pathogens, and parasites”) had become numerically dominant. Again, at day 7 the values were intermediate. Genes significantly regulated at day 3 included genes responsive to hypoxia and oxidative stress *Hif1α* (3.48), the antihemophilic Von Willebrand factor (*Vwf*; −3.09), a suppressor of apoptosis thymoma viral proto-oncogene 1 (*Akt1*; 2.31), the angiogenesis-related Thrombospondin 1 (*Thbs1*; 3.41), and DNA repair antiapoptotic Heat shock 70 kDa protein 1B (*Hspa1b*; −2.09). Genes significantly regulated at day 14 included an initiator of apoptosis *BCL-3* (46.57), a suppressor of apoptosis *Akt1* (−7.52), *Hif1*α (−3.37), and members of the complement cascade, including complement component 1, q subcomponent, alpha (*C1qa*; 2.37), complement component 1, q subcomponent, beta (*C1qb*; 4.56), complement component 1, q subcomponent, gamma (*C1qg*; 6.43), Complement component 3 (*C3*; 10.42), and Complement component 3 (*C4*; 5.41)

### Apoptosis-related genes

**Figure 4:**
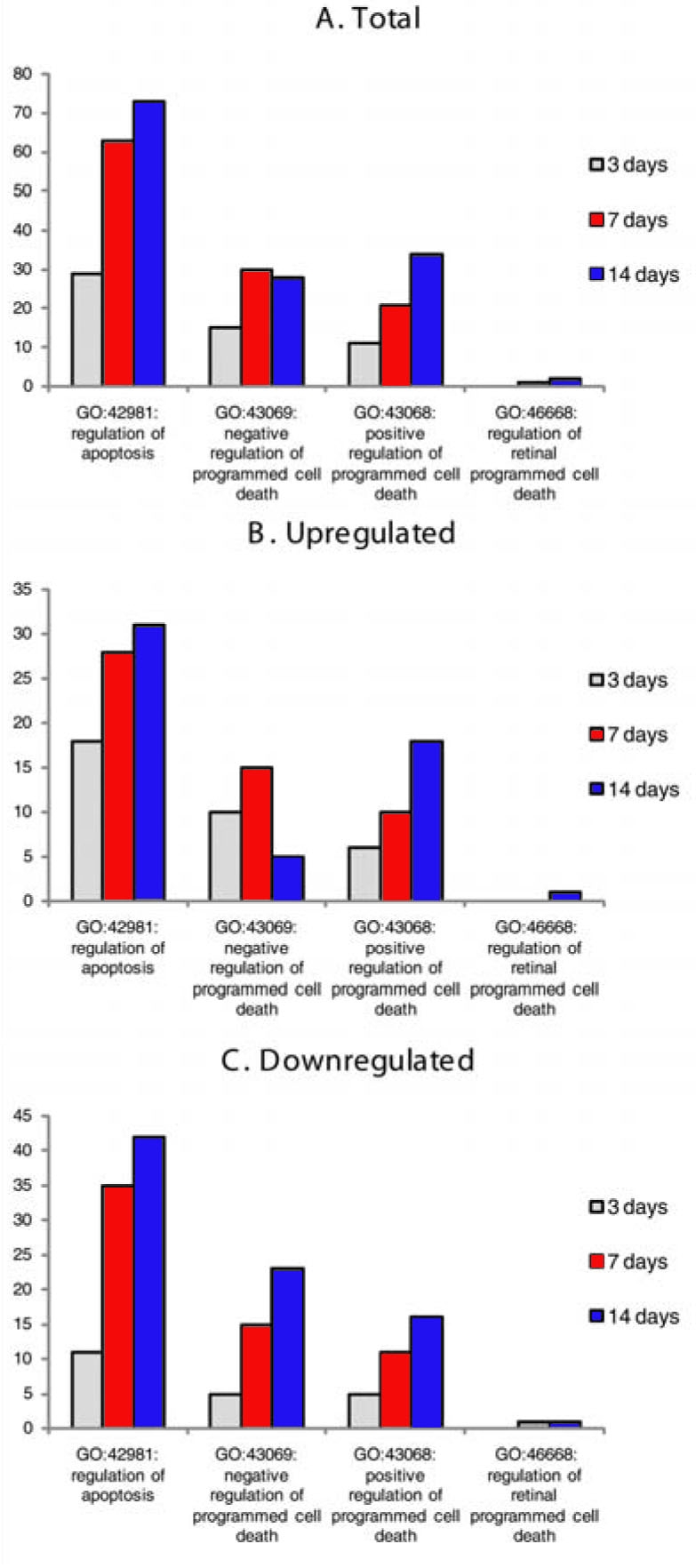
Number of regulated genes, total, upregulated, and downregulated, is shown by Gene Ontology at 3, 7 and 14 days. The total number (**A**) of apoptosis-related genes is maximal at 14 days for all subgrouping except for ‘negative regulation of programmed cell death’ which peaks at 7 days. The number of upregulated (**B**) and downregulated (**C**) genes increases over the 14 day treatment period in each ontology category except in (**B**) where ‘negative regulation of programmed cell death’ is maximal at 7 days. This indicates a loss in potential protective mechanisms against apoptosis in the hyperoxic mouse retina.

Significant trends in regulation were also apparent among genes related to apoptosis (Figure 4). First, the overall number of apoptosis-related genes whose expression was oxygen-regulated increased with exposure, including both death-promoting and death-inhibiting genes (Figure 4A). Second, when increases and decreases in expression were separated (Figure 4B,C), then at day 14, when photoreceptor death was prominent, the number of apoptosis inhibitors whose expression was increased fell; the number of apoptosis promoters whose expression was increased rose sharply. That is, the machinery of cell death had become predominant, within the overall increase shown in Figure 4A. Among the death promoter genes that became prominent at day 14 exposure were B-cell leukemia/lymphoma 3 (*Bcl3*), Bcl2 interacting killer-like (*Biklk*), Caspase 7 (*Casp7*). Prominent among the death-inhibitor genes whose expression fell at day 14 were apoptosis inhibitor 5 (*Api5*), insulin-like growth factor 1 (*Igf1r*) and *Akt1*.

### Pathways analysis of stress response

To identify stress-related pathways significantly regulated by hyperoxia, we used GO (Figure 4) to analyze the response-to-stress genes identified as regulated by hyperoxia using Database for Annotation, Visualization and Integrated Discovery (DAVID). This analysis identified two stress-related pathways as significantly regulated: 1) the “Inactivation of Gsk3 by AKT causes accumulation of β-catenin in Alveolar Macrophages (BIOCARTA) pathway,” which is important in macrophage activation; and 2) the Complement and Coagulation Cascades (KEGG) pathway (KEGG #: mmu04610).

Regulation of the “Inactivation of Gsk3 by AKT causes accumulation of β-catenin in Alveolar Macrophages pathway” was evident at all three periods of exposure to hyperoxia examined. At day 3, *Akt1* (2.31), toll interacting protein (*Tollip*; 3.65), Gap junction protein, alpha 1, (*Gja1*; 2.29) all increased expression, while at day 7 regulated genes included *Akt1* (2.00), *Gja1* (4.50), and Glycogen synthase kinase 3 beta (*Gsk3b*; −2.13) and at day 14, adenomatosis polyposis coli (*Apc*; −6.99), *Akt1* (−7.52), *Gja1* (2.37), *Tollip* (−2.17), lymphocyte antigen 96 (*Ly96*; −3.60), and *Gsk3b* (−2.04). One of the key roles of this pathway is the action of *Akt1* in inhibiting apoptosis, through interactions with NFκB; *Akt1* was upregulated at day 3 and day 7, but downregulated at day 14. Another key role is the interaction of *Gsk3b* and *Apc*, two of the three components (the third being axin) required for phosphorylation of β-catenin. This phosphorylation targets β−catenin for degradation, reducing its effect in inhibiting apoptosis.

Regulation of genes involved in the Complement and Coagulation Cascade pathway was significant principally at day 14 exposure to hyperoxia, the regulated genes including *C1qa* (2.37), *C1qb* (4.56), *C1qg* (6.43), *C3* (10.42), *C4* (5.41), *Serpinc1* (21.65), and *Serping1* (5.26). None of these genes was regulated significantly at shorter exposure times. Outcomes of the complement cascade include cell lysis, phagocyte recruitment, and inflammation.

## Discussion

Hyperoxia is an established feature of the retina undergoing photoreceptor degeneration. In retinitis pigmentosa, the effect of raised oxygen levels is apparent as a slowly progressive thinning of the retinal vasculature. In animal models, the increase in tissue oxygen levels in the photoreceptor layers caused by degeneration has been measured directly [14–16]; a comparable thinning of vessels has been observed and has been shown to be reversed by hypoxia. Further, hyperoxia is toxic to photoreceptors [9–13], raising the possibility that depletion-induced hyperoxia contributes to the progress of the retinal degenerations. This study is the first to provide an overview of gene expression changes induced in the retina by sustained hyperoxia.

### Summary of findings

The total number of genes showing altered (upregulated or down-regulated) expression increased in response to hyperoxia in the C57BL/6J mouse retina during a 14 day exposure period. At day 3, the numbers of genes increasing or decreasing expression were approximately equal (509 versus 688); by day 14, the number of regulated genes had tripled, and those decreasing expression had become numerically dominant (933 increasing versus 2169 decreasing). A hierarchical clustering analysis confirmed this change as progressive and consistent, with inconsistency greatest (suggesting a transition point) at day 7 exposure.

Genes in which mutations were known to cause photoreceptor dystrophy (RetNet database) were hyperoxia-regulated only at the day 14 exposure time point. Of these, 15 were identified (Table 4).

When stress-related genes were considered separately, the number regulated increased with time of exposure to hyperoxia, to be maximal at day 14. Within that overall pattern, however, there was a significant shift with time, with DNA-damage-related genes numerically dominant at day 3, and genes relating to tissue damage (wounding; pests, pathogens, and parasites) numerically dominant at day 14.

When apoptosis-related genes were considered separately, the number of genes regulated increased with time of exposure, to be maximal at day 14. Within that overall increase, again, there was a significant shift with time, with apoptosis-inhibiting genes dominant at day 3, and apoptosis-promoting genes dominant at day 14.

Finally, among the stress-related genes, pathway analysis identified significant regulation of two signaling pathways: one related to macrophage activation, and one to the regulation of the complement cascade.

### Phases in the retina’s response to hyperoxia

Previously [18], we have argued that the response of the C57BL/6J retina to sustained hyperoxia comprises successive early and late phases. In the early phase, at day 3 exposure, protective mechanisms are prominent, while the later stage (day 14) is dominated by the response to tissue damage.

The present results support this two-phase analysis in several ways. In the hierarchical clustering analysis, for example, cladistic separation was maximal between the day 3 and day 14 data; the day 7 replicates showed greater internal separation than the replicates at the other three time points. As previously reported [18] the day 7 time point in this model appears to be transitional. The shifts noted above between day 3 and day 14 in the expression of photoreceptor dystrophy genes (regulated only at day 14), of stress-related genes (from DNA-related to damaged-related), of apoptosis-related genes (from inhibiting to promoting) all support this idea of early and late phases. Relating these changes to retinal structure, we find photoreceptors remained stable at day 3 exposure, show signs of degeneration at day 7 and show considerable loss and continuing cell death at day 14.

Relating this pattern to individual genes, it is evident that at day 14 the genes responsible for the early stability of the retina were switched off, and genes that cause instability are switched on. For example, the microarray data indicated that *Hif1α*, *Akt1,* and *Oxr1* were upregulated at day 3, and downregulated by day 14; conversely, *BCL-3*, *Gadd45b*, *C3*, *Lin7b*, and *Edn2* were all upregulated late in the 14-day period examined.

### Functional roles of early- and late-responsive genes: examples

*Hif1α*, identified by the functional annotation tool of GeneSpring as a response-to-stress gene, is an established hypoxia-induced transcription regulator, thought to regulate the expression of at least 40 genes [21]. The genes regulated by *Hif1α* play a pivotal role in tissue adaptation changes in their local oxygen and metabolic environment. The evidence presented here suggests that *Hif1α* is regulated by hyperoxia, as well as hypoxia, and act during the early stages of hyperoxia (three days), before photoreceptor damage.

*Akt1* is identified by GeneSpring as being both stress- and apoptosis-related. It is a protein kinase known to initiate NF-κβ, an important proinflammatory transcription factor, and has been shown to protect hippocampal neurones from hypoxia- and nitric oxide-mediated cell death [22]. AKt1 has been found to be upregulated in response to retinal injury, and appears to be part of a neuroprotective response [23]. Akt1 protein is known to interact with BCL-3 protein, preventing its phosphorylation through inhibition of Gsk3, and slowing its subsequent degradation [24]. *BCL-3* is an oncogene which functions as a nuclear transcriptional activating factor that activates NF-κβ target genes. *Akt1*, Gsk3, and *BCL-3* have been implicated in the pathway “Inactivation of Gsk3 by AKT causes accumulation of b-catenin in Alveolar Macrophages.”

Little is known of the role of *Gadd45b* or *Lin7b* in the retina. *Gadd45b* responds to environmental stresses by mediating activation of the p38/JNK pathway [25,26] but has not yet been studied in the central nervous system. It may [27] act as an antiapoptotic gene regulated by NF-κβ. *Lin7b* is a polarity-related gene, and has been identified as a interactive partner of Rhotekin (an effector of Rho) and playing a role in neuronal function [28]. *Lin7b* might be involved in synapse function as it has been found to associate with the neuronal cadherin-β-catenin complex [29]. Increases in both *Gadd45b* and *Lin7b* at day14 indicate a potential role in neuronal survival.

*Edn2* and *GFAP* may function as general stress response genes in the retina. *Edn2* is a member of a vasoconstrictor family of genes and has been found to signal Müller cells in the stressed retina by binding to EDNRB [30]. GFAP is a stress-induced intermediate filament protein expressed by astrocytes and, under stress, by Müller cells [31,32]. Upregulation of these genes at day 14 confirms the stress caused by hyperoxia. Further work, specific to individual genes, will be required to define their roles more clearly.

### Hyperoxia-regulated retinal disease genes: examples

The present study provides evidence that hyperoxia regulates 15 genes in which mutations are known to cause photoreceptor dystrophy. For example, DMD is an X-linked recessive neuromuscular disease affecting skeletal, cardiac and smooth muscle function as well as the central nervous system. In the retina, mutations in DMD lead to reduction in rod b-wave amplitude but normal a-wave, photoreceptor morphology and visual acuity [33]. This suggests that a mutation in DMD causes a breakdown in retinal processing of photoreceptor signals.

*C3* forms part of the complement cascade and has been implicated, along with other proteins of the cascade, in several inflammatory diseases including age-related macular degeneration and Alzheimer disease [34–36]. In this study, genes for proteins in the classical (*C1qa*, *C1qb*, *C1qg*, *C4*, and *Serping1*) and alternative cascade (*C3*) have been shown to be upregulated at 14-day exposure to hyperoxia. *C3* has been found to be expressed in RPE cells, and accumulation of C3 protein has been found in a knockout of CFH^−/−^ mouse, in the photoreceptor outer segments [37]. Mutations in CFH cause accumulation of C3 in RPE cells, and it is this accumulation that leads to inhibition of transduction and possibly cell death [37]. It is possible that hyperoxia causes a comparable accumulation of C3.

### Paradox: how does hyperbaric oxygen treatment slow retinal degenerations

It seems counter to the evidence discussed above of the toxicity of hyperoxia to photoreceptors that hyperbaric oxygen therapy (HBO) has been reported to improve the ERG in humans affected by retinitis pigmentosa, and to slow the progress of the degenerations [38]. In HBO, the retina (indeed the whole body) is exposed to very high partial pressures of oxygen achieved by exposing the patient to 100% oxygen under 2–3 atmosphere of pressure (atm). Although hemoglobin is close to saturated in normobaric normoxia (21% oxygen at 1 atm), the partial pressure of oxygen in body fluids is increased several-fold in HBO. Typically, HBO is given for 1–2 h daily, for 5 days per week. In the trials reported by Vingolo and colleagues, patients were given 100% oxygen at 2.2 atm for 90 min, 3 times a day and 5 days a week for a month, then for 5 consecutive days monthly for 11 months, then for 5 consecutive days every 3 months for up to 9 years.

The early-late phase analysis of the C57BL/6J retina’s response to hyperoxia gives a clue as to why HBO may provide benefit to some patients. Because exposure times are short (1–2 h) compared to the early and late phases discussed here (measured in days), it is possible that HBO elicits the early phase genes by repeated, successive episodes of hyperoxia. Further experimentation is needed to test this possibility; if confirmed, this suggestion would provide a mechanistic hypothesis for the beneficial effects of the extreme oxygen tensions generated by repeated episodes of HBO.

## Appendix 1. Genes identified as hyperoxia regulated using ANOVA

To access the data, click or select the words “Appendix 1.” This will initiate the download of a pdf file archive that contains the file.

The genes listed were identified using ANOVA as hyperoxia-regulated by two criteria: a greater than twofold change and a p value < 0.05. The genes highlighted in red were examined by qPCR to validate the GeneChip® results. These include *BCL-3*, *C3*, *GFAP*, *Edn2*, *Lin7b*, and *Gadd45b*. Increases are highlighted in black bold, and decrease are in normal text. NC=No change.
